# Structure and spectral properties of Dy^3+^ doped CaYAlO_4_ single crystal

**DOI:** 10.1038/s41598-023-33366-x

**Published:** 2023-04-13

**Authors:** Yunyun Liu, Yan Wang, Meng Wang, Huan Shen, Chuanxin Huang, Xihu Wang, Ju Gao, Chaoyang Tu

**Affiliations:** 1 School of Opto-Electronic Engineering, Zaozhuang University, Zaozhuang, 277160 Shandong China; 2 Key Laboratory of Functional Materials and Devices for Informatics of Anhui Educational Institutions, Department of Physics, Fuyang Normal University, Fuyang, 236037 Anhui China; 3 Key Laboratory of Optoelectronic Materials Chemistry and Physics, Fujian Institute of Research on the Structure of Matter, Chinese Academy of Sciences, Fuzhou, 350002 Fujian China

**Keywords:** Lasers, LEDs and light sources, Optical materials and structures

## Abstract

A 2 at.% Dy^3+^: CaYAlO_4_ single crystal was grown successfully. The electronic structures of Ca^2+^/Y^3+^ mixed sites in CaYAlO_4_ were investigated using first-principles based on density functional theory. The effects of Dy^3+^ doping on the structural parameters of host crystal were studied using XRD pattern. The optical properties including absorption spectrum, excitation spectrum, emission spectra and fluorescence decay curves were thoroughly investigated. The results show that the Dy^3+^: CaYAlO_4_ crystal could be pumped by the blue InGaN and AlGaAs or 1281 nm laser diodes. Furthermore, an intense 578 nm yellow emission was obtained directly under excitation at 453 nm, meanwhile, evident mid-infrared light emitting was observed by 808 or 1281 nm laser excitation. The fitted fluorescence lifetimes of ^4^F_9/2_ and ^6^H_13/2_ levels were about 0.316 ms and 0.038 ms, respectively. It can be concluded that this Dy^3+^: CaYAlO_4_ crystal could simultaneously act as a promising medium for both solid-state yellow and mid-infrared laser outputs.

## Introduction

570–590 nm visible lasers and 3–5 µm mid-infrared (MIR) lasers have attracted much attention due to their important applications in various fields, especially in the medical field, such as the treatment of skin and eye diseases^1–5^. Therefore, the development of spectral 570–590 nm yellow lasers and 3–5 µm MIR lasers is of great significance. Especially for yellow lasers, nonlinear frequency conversion is still the most popular technique for obtaining yellow lasers^6,7^, which belongs to indirect method. However, the above-mentioned technology for indirectly obtaining yellow laser is expensive, inefficient and complex. These drawbacks inspire researchers to develop a new method for obtaining yellow lasers. Recently, due to the rapid development of laser diode (LD) technology, a simpler and more reliable system for the direct generation of yellow lasers has been proposed, which is achieved by LD-pumping rare-earths doped laser materials^8,9^. Therefore, materials capable of directly emitting yellow lasers have received great attention.

Trivalent dysprosium (Dy^3+^) ions are widely used in W-LEDs and MIR lasers due to their unique luminescence characteristics in the visible light regions of 460–500 nm (^4^F_9/2_ → ^6^H_15/2_), 550–600 nm (^4^F_9/2_ → ^6^H_13/2_), and the MIR regions of 2400–3500 nm (^6^H_13/2_ → ^6^H_15/2_) and 3700–4800 nm (^6^H_11/2_ → ^6^H_13/2_)^10–16^. However, the concentration quenching effect (CQE) of Dy^3+^ is the major issue^17^. Despite this, Dy^3+^-doped laser materials, such as Dy: YAG^18,19^, Dy, Tb: Y_3_Al_5_O_12_^20^, Dy: LiLuF_4_^21^, Dy: ZnWO_4_^9^, Dy: LaF_3_^22^, Dy: CaF_2_^23^, have yielded promising results in yellow lasers and 3–5 µm MIR lasers. Also, more meaningful achievements in this field are expected in the near future.

CaYAlO_4_ (CYA) crystal is regarded as a promising laser host material due to its spectral inhomogeneous broadening property, which is mainly due to its hybrid structure^24^. The CYA crystal belongs to the tetragonal system with space group *I4/mmm*. The crystal parameters are *a* = *b* = 3.6451 Å, *c* = 11.8743 Å^25^. The Yb^3+^, Nd^3+^, Pr^3+^, Ho^3+^ and Er^3+^-doped CYA crystals have been reported for its excellent physical and chemical properties^26–30^. However, reports on the use of Dy^3+^-doped CYA crystals to simultaneously produce yellow and MIR lasers are scarce so far.

As the CYA crystal melts congruently, a Dy^3+^-doped single crystal was successfully grown using the Czochralski technique. The crystal structure and electronic structures of the grown crystal were investigated. The luminescence properties of the grown crystal were also discussed using the measured spectral parameters.

## Experimental

A classical solid-phase sintering process was used to synthesize polycrystalline powder with the chemical formula of Dy_0.02_CaY_0.98_AlO_4_ (Dy: CYA). The chemical raw materials used were CaCO_3_, Al_2_O_3_ (AR grade) and Y_2_O_3_, Dy_2_O_3_ (4 N purity) powders. All of them were purchased from Changchun Heprui Rare Earth Materials Technology Co., Ltd. The crystal grown was carried out in a DGL-400 furnace (NCIREO, China). The crystal growth process and parameter settings are similar to those described in the Ref.^30^. Finally, a high-quality Dy: CYA crystal for this experiment was obtained.

The concentration of Dy^3+^ in the singly-doped CYA crystal was measured by inductively coupled plasma-atomic emission spectrometry (ICP-AES, Ultima 2, Jobin-Yvon). The result was 3.59 × 10^20^ ions cm^−3^. The segregation coefficient *k* of Dy^3+^ in the Dy: CYA crystal was calculated by

1 $$ k = c_{s} /c_{o} $$

where *c*_*s*_ and *c*_0_ were the concentrations of Dy^3+^ in the crystal and initial raw materials, respectively. The value of *k* was 1.42.

The CYA single crystal structure data used for theoretical calculations were obtained from the ICSD database (No. 1001545). The approximate process and parameter settings of the theoretical calculations are similar to those described in the Ref.^24^. Here, the cutoff energy is set to 380 eV. The *k*-point grids used for the unit cell geometry optimization and electronic structure calculation of the Brillouin zone are 3 × 3 × 1 and 7 × 7 × 2, respectively. Interactions between ionic cores and valence electrons are described, including Ca-3s^2^3p^6^4s^2^, Y-4s^2^4p^6^5s^2^4d, Al-3s^2^3p, and O-2s^2^2p^4^ electrons.

The powder XRD pattern of the grown crystal was performed on a Miniflex600 with a diffracted beam monochromator set for *Cu-Kα* radiation (λ = 1.54056 Å). The range of 2θ was 10°–80°, and the scan step size was 0.02°.

In addition, the absorption spectrum was recorded using a Perkin-Elmer UV–Vis–NIR Spectrometer (Lambda-900) in a range of 400–2000 nm. The fluorescence spectra and emission decay curves with different pump sources were measured using an Edinburgh Instruments FLS920 Spectrophotometer. All spectra were measured at room temperature (RT). The dimensions of the experimental sample used for spectral testing were 10.0 × 8.0 × 1.0 mm^3^.

## Results and discussion

### Crystal structure and electronic structures

**Figure 1:**
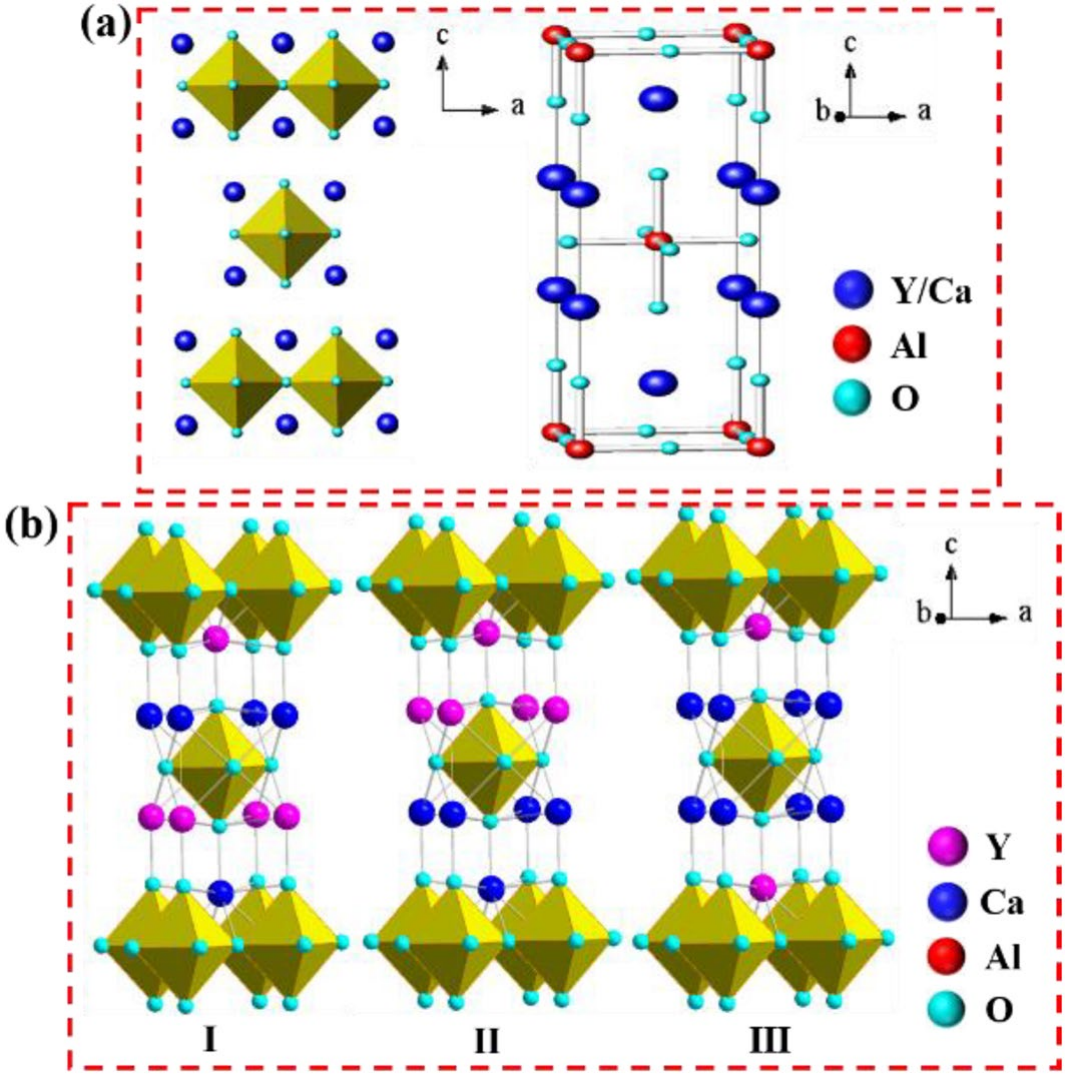
(**a**) The crystal structure model and (**b**) three nonequivalent configurations of the CYA unit cell (denoted as “I”, “II” and “III”).

As a member of the ABCO_4_ family, CYA has a perovskite-type structure with Al ions occupying octahedral symmetry sites and divalent Ca and trivalent Y randomly distributed in a 1:1 ratio at *C*_4*v*_ symmetry sites, as shown in Fig. 1a. Due to the unique properties of Ca^2+^ and Y^3+^, the perfect unit cell can be divided into three non-equivalent configurations, as shown in Fig. 1b. In addition, due to their similarity in radius and valence, Y^3+^ are easily replaced by Dy^3+^, which further increases the disorder of the crystal. This can cause non-uniform broadening of absorption and emission lines. The broad absorption spectrum is beneficial for increasing the absorption of pump light, and the broad emission spectrum is conducive to obtaining a tunable or ultrafast laser^31^.

**Figure 2:**
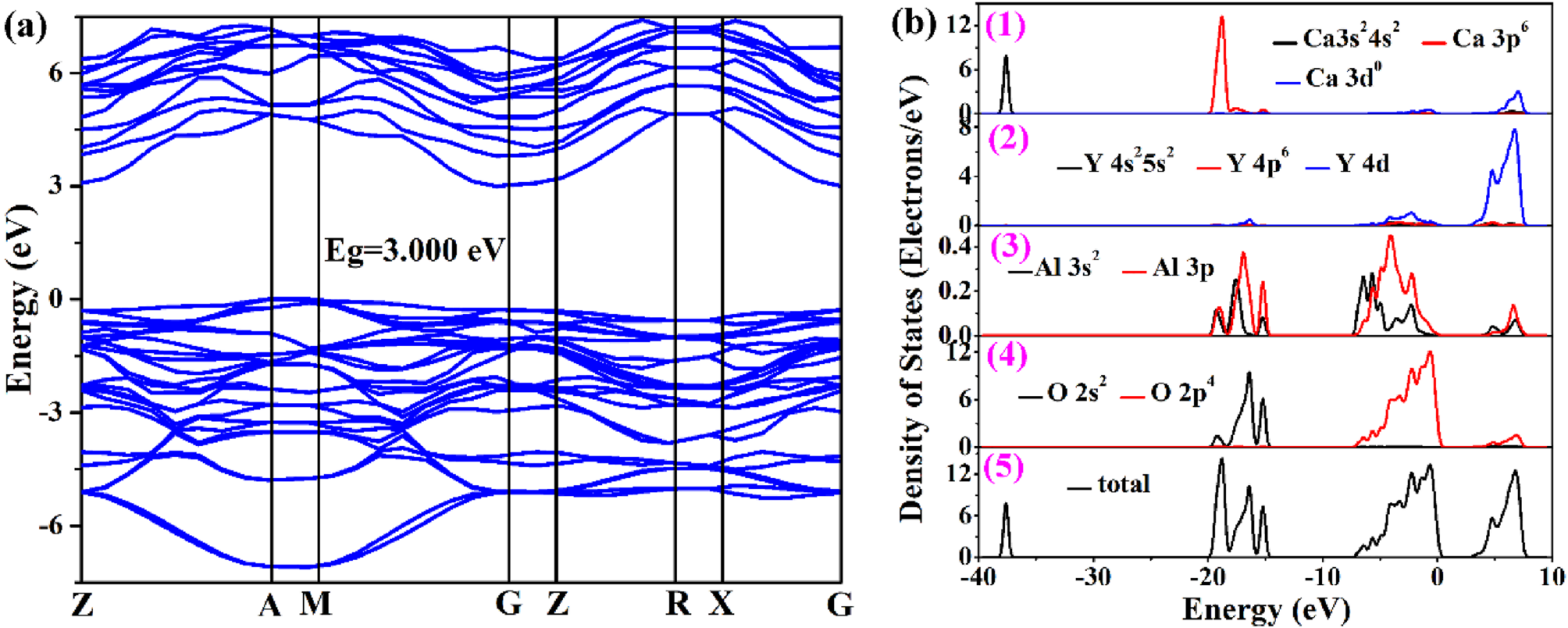
(**a**) Calculated band structure of CYA; (**b**) electronic DOS of CYA: (1)–(4) the partial DOS of Ca, Y, Al, O and (5) the total DOS.

To understand the bonding interactions in CYA, theoretical calculations based on the DFT method were performed. Figure 2a demonstrates the band structure of CYA (configuration “I”), which shows that the CYA is a kind of direct band gap material with a bandgap of 3.000 eV. For the unit cell of configurations “II” and “III”, the band structures of CYA are also calculated and shown in Fig. S1(a) and Fig. S2(a), respectively.

Figure 2b shows the full and partial density of states (DOS) (configuration “I”), which contributes to the bands. Since the CYA single crystal is composed of AlO_6_ octahedron, the valence band is mainly provided by the Al-O bond. For the conduction band, from 7.8 to 0 eV, the O-2p^4^, Al-3s^2^, 3p, Y-4d, and Ca-3d^0^ states are mainly involved. For the other two configurations, the corresponding densities of states are shown in Fig. S1(b) and Fig. S2(b), respectively.

### X-ray and Rietveld refinement analysis

**Figure 3:**
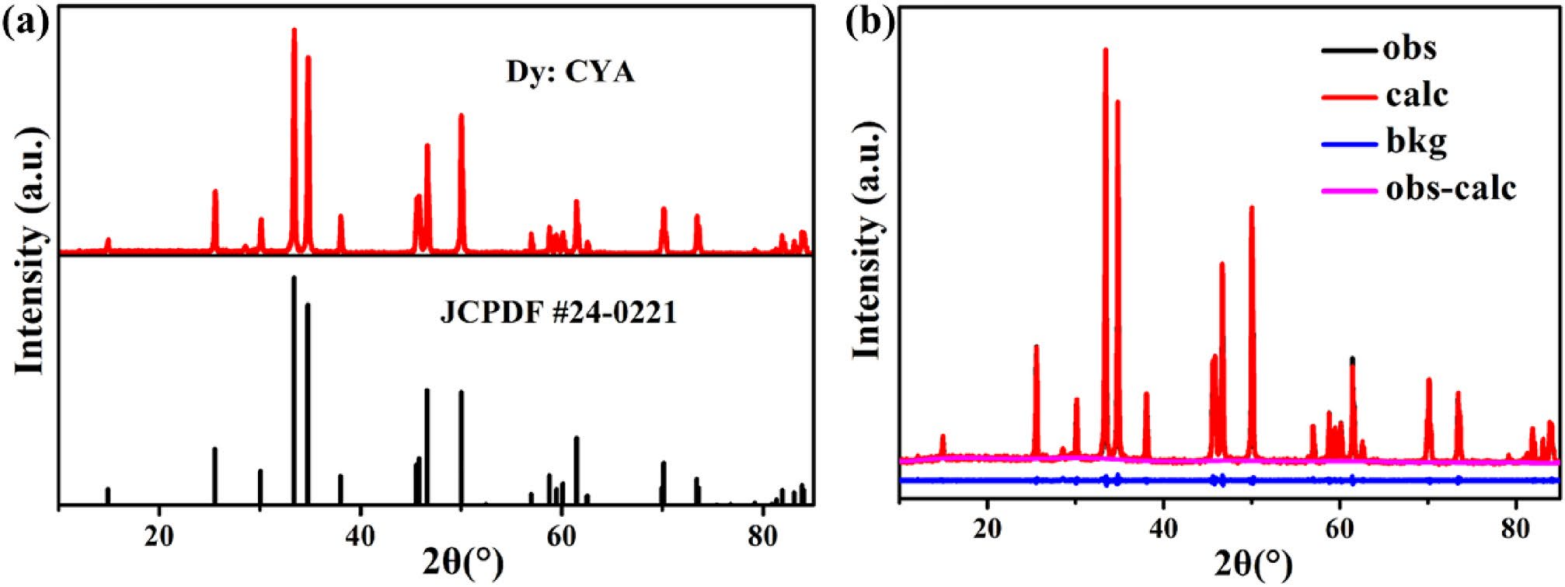
(**a**) XRD pattern of Dy: CYA crystal; (**b**) the refined results of Dy: CYA crystal (the "bkg" refers to the background diffraction peak intensity. The "obs" refers to the experimentally measured raw data, "cal" refers to theoretical simulation data, and "obs-calc" refers to the difference between the two).

The measured XRD pattern confirmed the phase purity and crystallinity of Dy^3+^-doped CYA crystal, as shown in Fig. 3a. The diffraction peaks of the sample are consistent with those of the standard JCPDF file [No. 24–0221], which indicates that Dy^3+^ were successfully introduced into the CYA host lattice. The structure of the Dy: CYA crystal is further refined using XRD data. The related refinement results and structural parameters are shown in Fig. 3b and Table 1, respectively. As shown in Fig. 3b, the observed and calculated diffraction patterns are consistent, indicating that the synthesized Dy: CYA crystal still has a tetragonal phase with space group *I4/mmm*. Furthermore, as can be seen in Table 1, the parameters of the Dy: CYA crystal become slightly smaller compared to JCPDF #24-0221 (a = b = 3.648 Å, c = 11.890 Å), which is mainly caused by the inconsistent radius between doped ions and Y. The radius of the dopant ion Dy^3+^ (0.908 Å) is smaller than that of Y^3+^ (1.075 Å).

**Table 1:** The parameters of refined structure.

Parameters	Dy: CYA
Crystal structure	Tetragonal
Space group	*I4/mmm*
a = b (Å)	3.64730 ± 0.00029
c (Å)	11.88342 ± 0.00029
*α* = *β* = *γ*	90°
*R*_*P*_	6.01%
*R*_*WP*_	9.04%

### The absorption spectrum and Judd–Ofelt analysis

**Figure 4:**
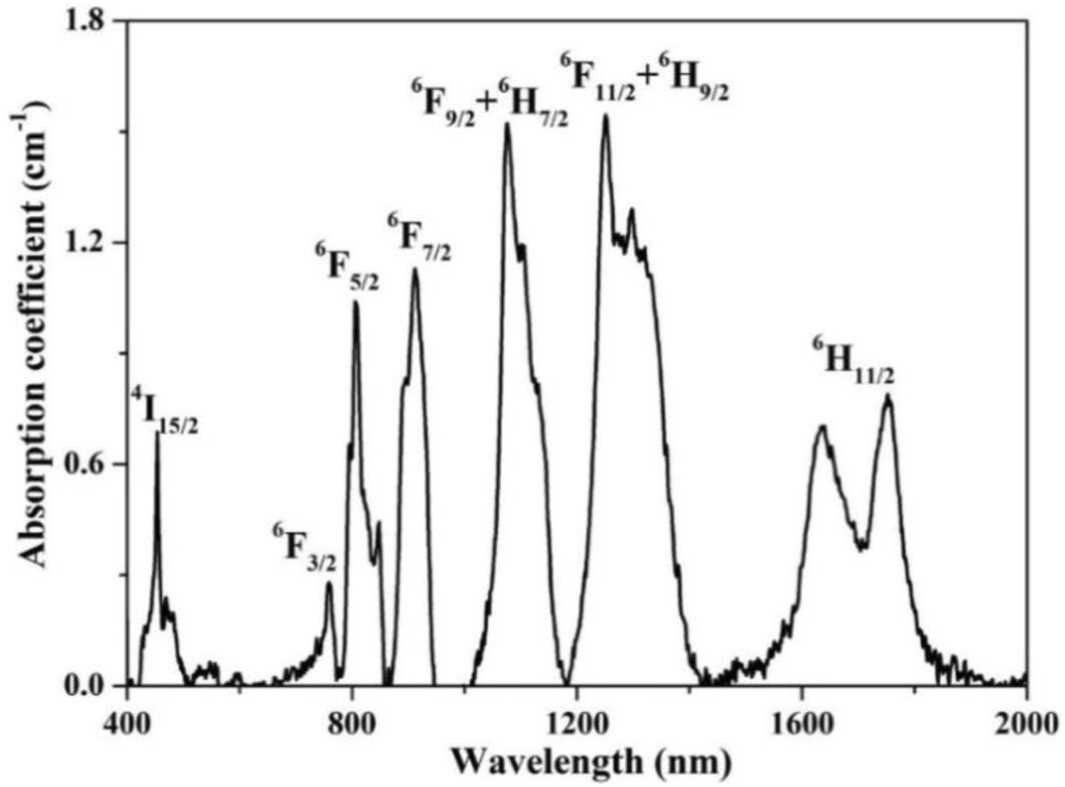
Absorption spectrum of Dy: CYA crystal.

**Figure 5:**
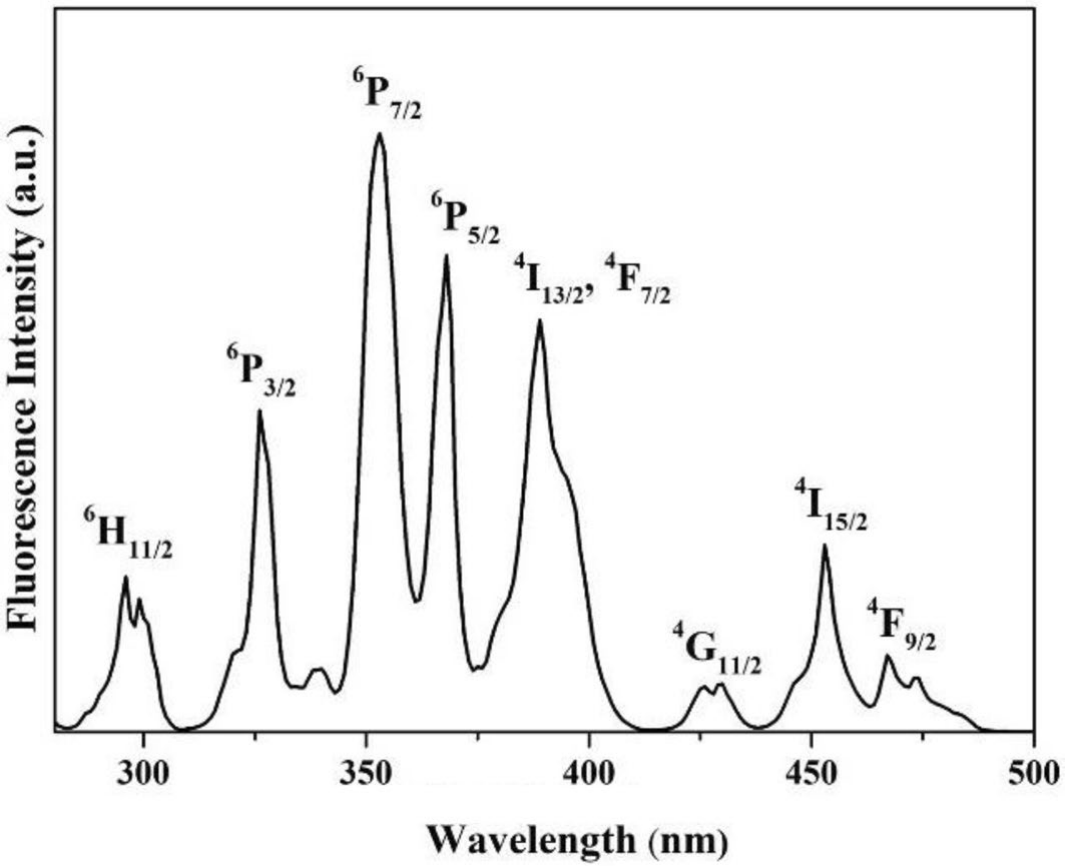
The excitation spectrum of Dy: CYA crystal.

Figure 4 illustrates the absorption spectrum of Dy: CYA crystal in the 400–2000 nm range. There are seven prime absorption bands located at 453, 758, 806, 909, 1076, 1251, and 1633 nm, that correspond to the transitions of the Dy^3+^ from the ground state energy level ^6^H_15/2_ to various excited states, as marked in Fig. 4. The absorption bands corresponding to the transitions of ^6^H_15/2_ → ^4^I_15/2_, ^6^H_15/2_ → ^6^F_5/2_, and ^6^H_15/2_ → ^6^F_11/2_ + ^6^H_9/2_ piqued our interest, because their peaks at 453, 808 and 1281 nm coincide with the output wavelengths of the commercial LDs. As shown in Fig. 5, the peaks of Dy^3+^: ^4^I_15/2_ energy level overlaps with the emission of blue InGaN LD. Therefore, yellow solid-state laser pumped by blue LD can be made via the transition of Dy^3+^: ^4^F_9/2_ → ^6^H_13/2_. Furthermore, the peaks of Dy^3+^: ^6^F_5/2_ and ^6^H_9/2_&^6^F_11/2_ energy levels overlaps with the emission of AlGaAs and 1280 nm LDs, respectively. Consequently, the 808 nm and 1281 nm LD pumped MIR laser can be made based on Dy^3+^: ^6^H_13/2_ → ^6^H_15/2_ transition (as shown in Fig. 9).

As an important spectral parameter, known as the absorption cross-section (*σ*_*a*_) can be determined by the following formula:

2 $$ \sigma_{a} \left( \lambda \right) = \frac{2.303}{{N_{0} \times l}}OD\left( \lambda \right) $$

where* λ* is the wavelength, *N*_0_ is the Dy^3+^ concentration, *l* is the thickness of the crystal and *OD* is the optical density. The calculated *σ*_*a*_ values are 1.86 × 10^–21^ cm^2^, 3.14 × 10^−21^ cm^2^, and 4.32 × 10^–21^ cm^2^ for 453 nm, 808 nm, and 1281 nm, respectively.

The radiative transition of the *4f* configuration of Dy^3+^ in Dy: CYA crystal was analyzed using Judd–Ofelt (J–O) theory^32,33^ and measured absorption spectrum. The details of the J–O calculation method can be found in the literature^34^. The results of the calculation are listed in Table 2. It shows that the calculated oscillators are consistent with the measured oscillators. The reality and validity of the results can be evaluated by the root mean square deviation (RMS *Δf*). Here, the RMS *Δf* is calculated to be 0.08801 × 10^–6^, indicating that the calculated results have a very high reference value. Furthermore, three intensity parameters *Ω*_*t*_ (t = 2, 4, 6) are fitted to be 1.97 × 10^–20^ cm^2^, 1.56 × 10^–20^ cm^2^, and 2.51 × 10^–20^ cm^2^, respectively. In general, the *Ω*_2_ can reflect the coordination symmetry of matrix materials and the orderliness of structures, which is sensitive to component changes, while *Ω*_4_/*Ω*_6_ is the spectroscopic quality factor^35^. In comparison to other Dy^3+^-doped crystals, the value of *Ω*_4_/*Ω*_6_ in Dy: CYA crystal is 0.62, which is larger than that in PbF_2_, Lu_2_SiO_5_, YAG, GSAG and LiYF_4_ crystals, as shown in Table 3.

**Table 2:** Oscillator strengths of Dy: CYA crystal.

Transition ^6^H_15/2_ →	*λ* (nm)	*n*	*f*_exp_ (× 10^–6^)	*f*_cal_ (× 10^–6^)
^4^I_15/2_	453	1.931	0.534	0.510
^6^F_3/2_	760	1.896	0.200	0.251
^6^F_5/2_	807	1.893	1.217	1.333
^6^F_7/2_	911	1.889	2.564	2.723
^6^F_9/2_, ^6^H_7/2_	1098	1.884	3.187	3.143
^6^F_11/2_, ^6^H_9/2_	1293	1.880	3.952	3.965
^6^H_11/2_	1693	1.874	1.432	1.334
			*RMS Δf* = 0.08801 × 10^–6^

**Table 3:** J–O parameters of Dy^3+^-doped laser crystals.

Crystals	*Ω*_2_ (× 10^–20^ cm^2^)	*Ω*_4_ (× 10^–20^ cm^2^)	*Ω*_6_ (× 10^–2^ cm^2^)	*Ω*_4_*/Ω*_6_	References
PbF_2_	3.18	1.16	2.27	0.51	^36^
Lu_2_SiO_5_	4.31	1.28	3.49	0.37	^37^
YAl_3_(BO_3_)_4_	10.81	2.05	3.28	0.63	^38^
BaY_2_F_8_	1.52	2.33	3.67	0.63	^39^
YAG	1.49	0.94	3.20	0.29	^40^
GSAG	2.17	1.06	2.32	0.46	^41^
LiYF4	2.01	1.34	2.39	0.56	^42^
CYA	1.97	1.56	2.51	0.62	This work

The radiative transition rate *A*, fluorescence branching ratio *β*, and radiative lifetime *τ*_r_ of Dy^3+^ transits from ^4^F_9/2_ or ^6^H_13/2_ to different lower levels were calculated using the obtained *Ω*_*t*_ parameters and are listed in Table 4. For the ^4^F_9/2_ level, the ^4^F_9/2_ → ^6^H_13/2_ transition has the largest radiative transition rate and the fluorescence branching ratio (51%). The calculated results indicate that the Dy: CYA crystal has great potential for producing yellow and MIR laser outputs.

**Table 4:** Calculated spontaneous radiation probabilities, branching ratios, and radiative lifetime of Dy: CYA crystal.

Transition		*A*_*ed*_	*A*_*md*_	*A* (s^−1^)	*β*	*τ*_r_ (ms)
^4^F_9/2_ →	^4^F_5/2_	4.321	0		0.003	0.758
	^6^F_7/2_	6.827	9.038		0.012	
	^6^H_5/2_	5.662	0		0.004	
	^6^H_7/2_, ^6^F_9/2_	34.957	14.666		0.038	
	^6^F_9/2_, ^6^F_11/2_	39.57	62.250		0.100	
	^6^H_11/2_	48.878	18.961		0.051	
	^6^H_13/2_	672.486	0		0.510	
	^6^H_15/2_	370.951	0	1319	0.281	
^6^H_13/2_ →	^6^H_15/2_	36.486	13.038	49.523	1	20.192

### Yellow fluorescence spectra

Figure 5 shows the RT excitation spectrum for the emission at 582 nm. Eight main excitation peaks centered at 296, 326, 353, 368, 389, 429, 453, and 467 nm were observed, which corresponds to the transition from the ground level ^6^H_15/2_ to upper levels ^6^H_11/2_, ^6^P_3/2_, ^6^P_7/2_, ^6^P_5/2_, ^4^I_13/2_ + ^4^F_7/2_, ^4^G_11/2_, ^4^I_15/2_, and ^4^F_9/2_, respectively. Although the most intense peak is at 353 nm, the output power of 350 nm LDs is lower. Therefore, the blue GaInN LD at 453 nm was used as the pumping source.

**Figure 6:**
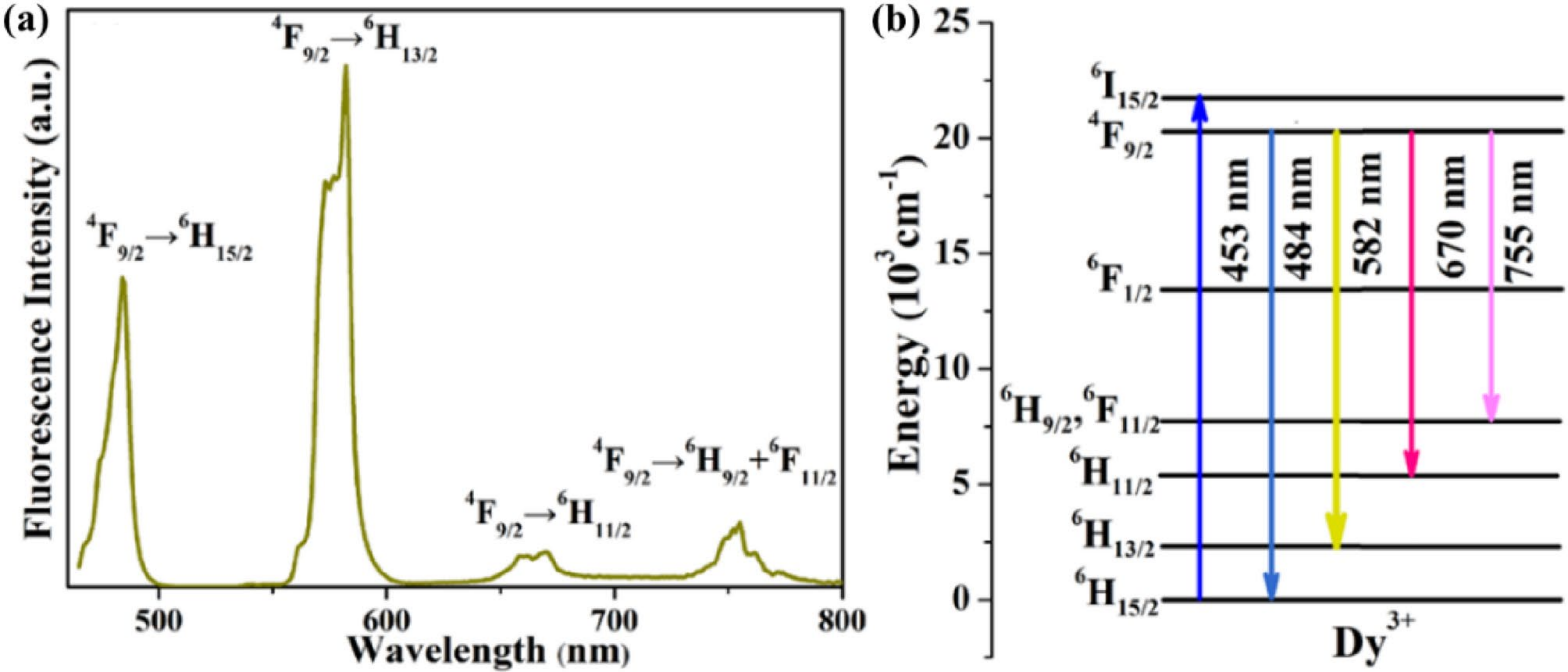
(**a**) The visible emission spectrum of Dy: CYA crystal; (**b**) the diagram of the corresponding energy level transitions.

Then the emission spectrum in the visible band excited by 453 nm was measured and presented in Fig. 6a. The emission bands centered at 484, 582, 670, and 755 nm can be seen in Fig. 6a, and the corresponding transition processes are shown in Fig. 6b. The strongest emission is concentrated at 582 nm, which is consistent with the fluorescence branching rate calculated by the J–O theory. The corresponding FWHM of ^4^F_9/2_ → ^6^H_13/2_ emission is 14.3 nm.

The stimulated emission cross-section can be calculated by the Füchtbauere–Ladenburg (F–L) formula^43,44^:

3 $$ \sigma_{em} = \frac{{\lambda^{5} A_{{JJ^{\prime}}} }}{{8\pi cn^{2} }}\frac{I\left( \lambda \right)}{{\smallint \lambda I\left( \lambda \right)d\lambda }} $$

where *I*(*λ*) is the experimental fluorescence intensity at wavelength *λ*. The value of *σ*_*em*_ at 582 nm is 0.24 × 10^–20^ cm^2^.

**Figure 7:**
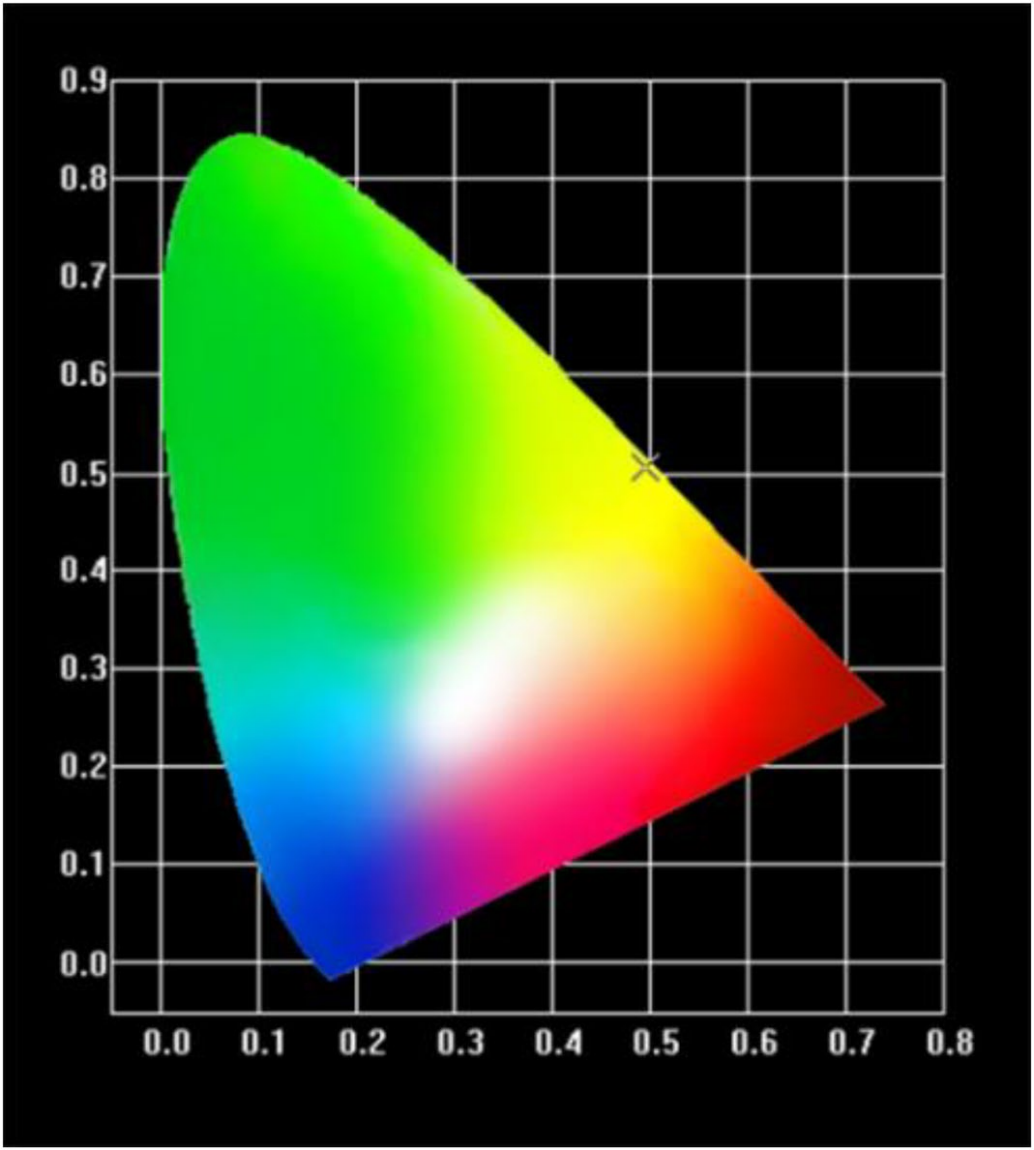
The chromaticity coordinate CIE 1931 of Dy: CYA crystal.

Moreover, the chromaticity coordinate CIE 1931 for Dy: CYA crystal was calculated and shown in Fig. 7. The obtained chromaticity coordinate is (x = 0.4946, y = 0.5044), which is in the yellow area. The correlated color temperature (CCT) can be calculated by the following formula^45^:

4 $$ {\text{CCT}} = - 449{\text{n}}^{3} + 3525{\text{n}}^{2} - 6823.3{\text{n}} + 5520.33 $$

here ${\text{n}} = ({\text{x}} - {\text{x}}_{{\text{e}}} )/({\text{y}} - {\text{y}}_{{\text{e}}} )$ and ${\text{(x}}_{{\text{e}}} {\text{, y}}_{{\text{e}}} {) = (0}{\text{.332, 0}}{.186)}$. The value of CCT was 2928 K. The above results indicate that the Dy: CYA can be used as a new material for yellow light emission.

**Figure 8:**
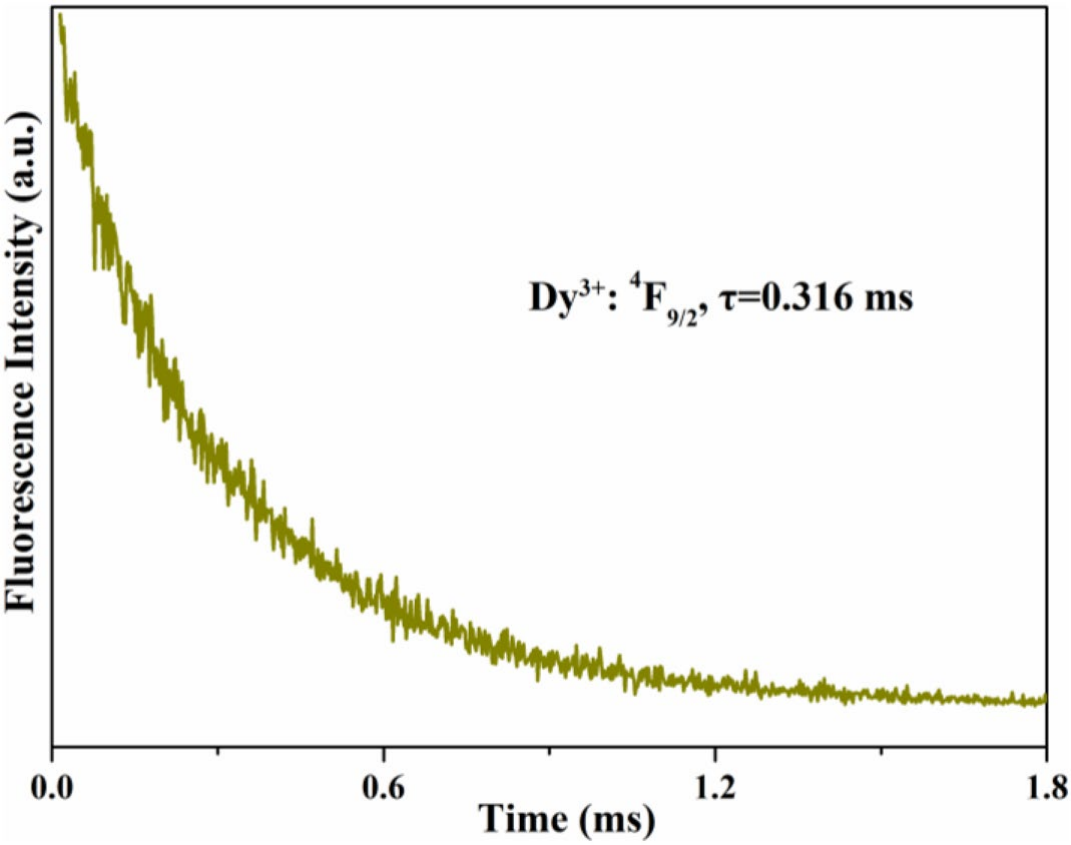
Fluorescence decay curve of the Dy^3+^: ^4^F_9/2_ multiplet.

Figure 8 shows the fluorescence decay curve of the Dy^3+^: ^4^F_9/2_ level pumped at 453 nm. This decay curve exhibits an exponential decay behavior. Therefore, the corresponding fluorescence lifetime can be fitted by the following formula:

5 $$ {\text{I}}({\text{t}}) = {\text{I}}_{0} \exp \left[ { - \frac{{\text{t}}}{\uptau }} \right] $$

where *τ* is the fluorescence lifetime. The fluorescence lifetime obtained by fitting is 0.316 ms. According to the equation: $\eta_{T} = \tau_{f} /\tau_{r} ,$ where $\tau_{r}$ is the radiative lifetime (as listed in Table 4), the quantum efficiency is about 41.6%.

Table 5 lists the main optical parameters of Dy: CYA and other Dy^3+^-doped crystals. For Dy: CYA crystal, the absorption cross-section at 453 nm is 1.86 × 10^–21^ cm^2^, which is larger than that of Dy: CaGdAlO_4_^46^, Dy: LiNbO_3_^47^, Dy: YAG^48^ and Dy: Li_2_Gd_4_(MoO_4_)_7_^49^. The emission cross-section at 582 nm is 0.24 × 10^–20^ cm^2^, which is larger than that of Dy: Li_2_Gd_4_(MoO_4_)_7_^49^. The fluorescence lifetime of Dy^3+^: ^4^F_9/2_ level is 0.316 ms, which is much longer than that of Dy: CaGdAlO_4_^46^ and Dy: LiNbO_3_^47^. The quantum efficiency is 48.90%, which is larger than that of Dy: CaGdAlO_4_^46^ and Dy: YAG^48^. The above-mentioned advantages indicate that the 2 at.% Dy: CYA crystal is a potential material for a yellow solid-state laser.

**Table 5:** The main optical parameters of some Dy^3+^-doped crystals.

Crystals	*σ*_*abs*_ (10^–21^ cm^2^)	*σ*_*em*_ (10^–20^ cm^2^)	*τ*_*f*_ (ms)	*τ*_*r*_ (ms)	*η *(%)	References
CaGdAlO_4_	2.43 (σ)	0.51 (σ)	0.222	0.501	44.31	^46^
	1.28 (π)	0.55 (π)				
LiNbO_3_	0.95 (σ)	0.03 (σ)	0.298	0.387	77.0	^47^
	0.6 (π)	0.32 (π)				
YAG	1.6	0.3	0.376	2.020	18.61	^48^
Li_2_Gd_4_ (MoO_4_)_7_	1.0 (σ)	0.17 (σ)	0.865	–	–	^49^
	1.4 (π)	0.16 (π)				
CYA	1.86	0.24	0.316	0.758	41.69	This work

### MIR emission spectra

**Figure 9:**
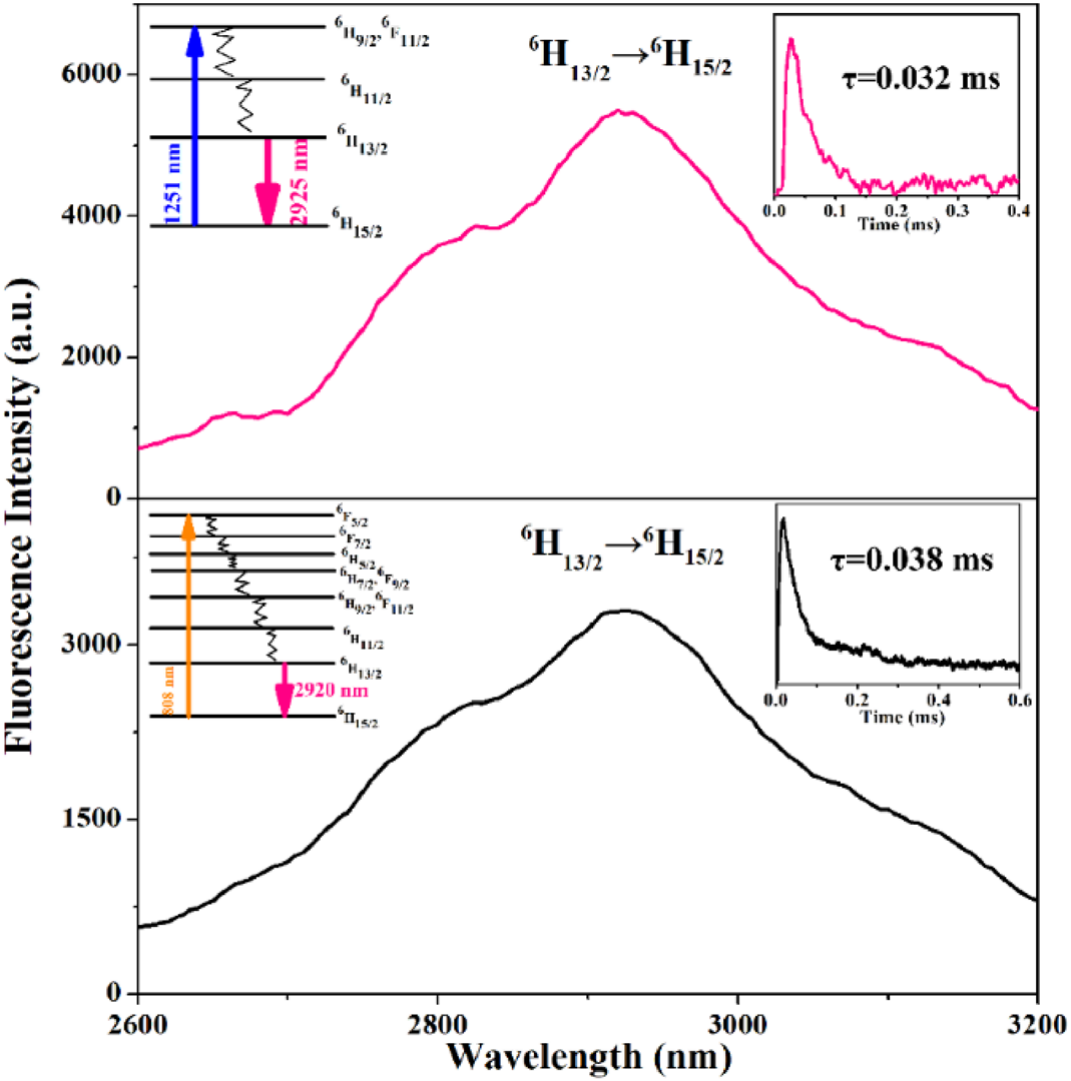
The MIR emission spectra and fluorescence decay curves of Dy^3+^: ^6^H_13/2_ in CYA crystal pumped by 1281 nm (upper panel) and 808 nm (lower panel).

The measured MIR emission spectra of Dy^3+^: ^6^H_13/2_ → ^6^H_15/2_, which were pumped by 1281 nm and 808 nm, are shown in Fig. 9. As shown in Fig. 4, it could be seen that the absorption band of ^6^H_15/2_ → ^6^H_9/2_/^6^F_11/2_ transition is very strong, which matches the emission band of the 1281 nm LD, so 1281 nm was chose for excitation wavelength, as shown in Fig. 9 (upper panel). In addition, the MIR emission spectrum with the pump wavelength of 808 nm was also analyzed and is shown in Fig. 9 (lower panel). According to the formula (3), when the crystal was excited by 1281 nm, the emission cross-section was 5.84 × 10^–21^ cm^2^ at the peak with an FWHM of 297 nm, otherwise it was 3.72 × 10^–21^ cm^2^ with an FWHM of 342 nm. Furthermore, the fluorescence lifetime *τf* of the Dy^3+^: ^6^H_13/2_ level was fitted to be about 0.032 ms for 1281 nm LD pumping and 0.038 ms for 808 nm LD pumping.

## Conclusion

Dy^3+^-doped CYA singly crystal was successfully grown. The electronic structures of CYA were presented and analyzed using first-principles calculations. The crystal lattice parameters were obtained by Rietveld refinement. The spectroscopic properties of the Dy: CYA crystal were investigated. According to the J–O theory, the evaluated intensity parameters are *Ω*_2_ = 1.97 × 10^–20^ cm^2^, *Ω*_4_ = 1.56 × 10^–20^ cm^2^ and *Ω*_6_ = 2.51 × 10^–20^ cm^2^. The main spectral parameters of the crystal are obtained and compared. An intense yellow emission was observed at 582 nm when 453 nm was used as a pump wavelength. The stimulated emission cross-section at the peak was 0.24 × 10^–20^ cm^2^. The fitted fluorescence lifetime of the ^4^F_9/2_ state was 0.316 ms. The color coordinate (CIE 1931) was also calculated (x = 0.4946, y = 0.5044), which was in the yellow area. When pumped by 1251 or 808 nm, a strong MIR emission was also observed at about 2920 nm and the stimulated emission cross-sections at peak were also calculated. The findings of this research indicate that the 2 at.% Dy: CYA crystal is not only a potential candidate for a blue InGaN LD directly pumped yellow laser, but it also has promising applications in the field of MIR luminescence.

## Supplementary Information

Supplementary Information.

## Data Availability

The datasets used and/or analyzed during the current study are available from the corresponding author on reasonable request.
