# Supplementary material for: Structure and spectral properties of Dy3+ doped CaYAlO4 single crystal
Source: Sci Rep. 2023 Apr 13;13:6066. doi: 10.1038/s41598-023-33366-x (PMC10101985; doi:10.1038/s41598-023-33366-x)
Supplement: Supplementary file 1 — Supplementary Information. [file 41598_2023_33366_MOESM1_ESM.docx]

**Supporting Information**

Structure and spectral properties of Dy^3+^ doped CaYAlO_4_ single crystal

Yunyun Liu^1*^, Yan Wang^3^, Meng Wang^1^, Huan Shen^3^, Chuanxin Huang^1^, Xihu Wang^2^, Ju Gao^1*^ & Chaoyang Tu^3^

^1^*School of Opto-Electronic Engineering, Zaozhuang University, Zaozhuang, Shandong, 277160, China*^2^*Key Laboratory of Functional Materials and Devices for Informatics of Anhui Educational Institutions, Department of Physics, Fuyang Normal University, Fuyang, Anhui, 236037, China*

*^3^Key Laboratory of Optoelectronic Materials Chemistry and Physics, Fujian Institute of Research on the Structure of Matter, Chinese Academy of Sciences, Fuzhou, Fujian, 350002, China*

^*^*E-mail: liuyunyun586@163.com*

^*^*E-mail: jugao@hku.hk*

**Contents**

Figure S1. (a) Calculated band structure of CYA; (b) Electronic DOS of CYA: (1)-(4) the partial DOS of Ca, Y, Al, O and the total DOS. (configuration “Ⅱ”)

Figure S2. (a) Calculated band structure of CYA; (b) Electronic DOS of CYA: (1)-(4) the partial DOS of Ca, Y, Al, O and the total DOS. **(**configuration “Ⅲ”**)**


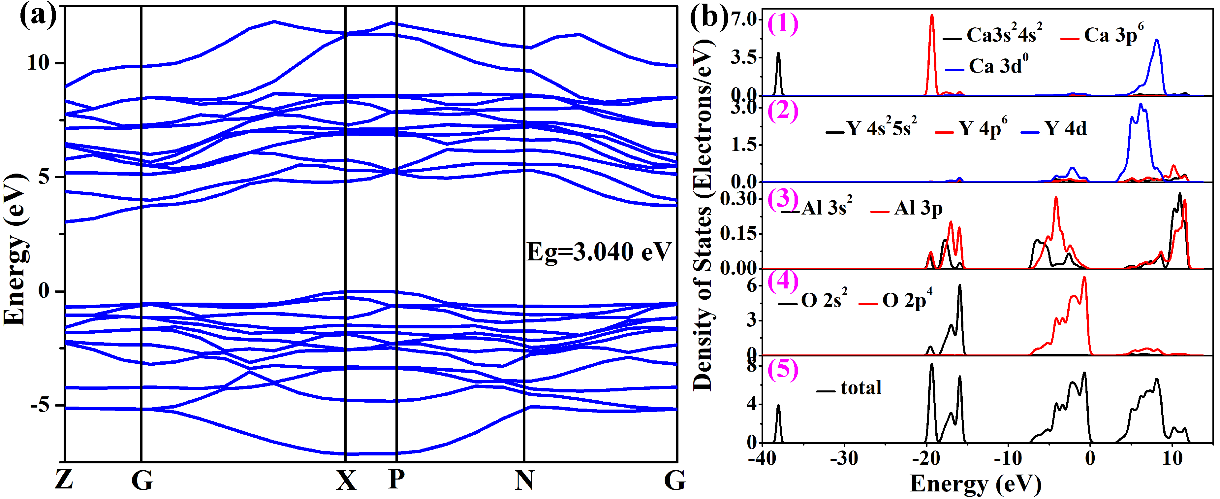


Figure S1. (a) Calculated band structure of CYA; (b) Electronic DOS of CYA: (1)-(4) the partial DOS of Ca, Y, Al, O and the total DOS. **(**configuration “Ⅱ”**)**


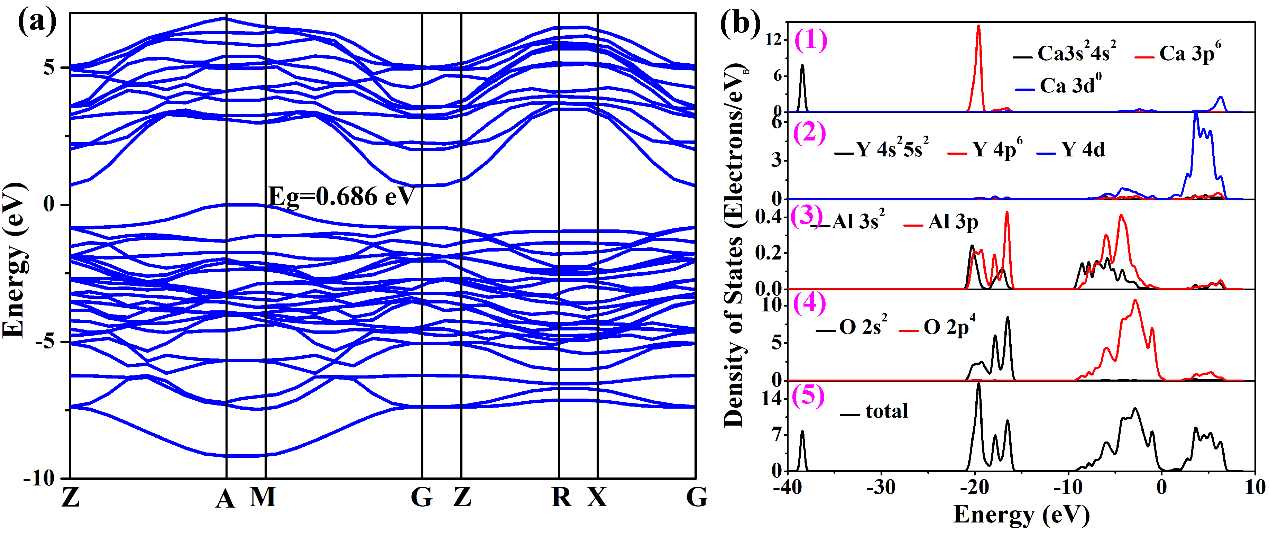


Figure S2. (a) Calculated band structure of CYA; (b) Electronic DOS of CYA: (1)-(4) the partial DOS of Ca, Y, Al, O and the total DOS. **(**configuration “Ⅲ”**)**
